# A retrospective analysis of health care utilization for patients with mitochondrial disease in the United States: 2008–2015

**DOI:** 10.1186/s13023-018-0949-5

**Published:** 2018-11-22

**Authors:** Bruce Cohen, Cristy Balcells, Brian Hotchkiss, Kavita Aggarwal, Amel Karaa

**Affiliations:** 1 Akron Children’s Hospital, Akron, OH USA; 2 MitoAction (former), Boston, MA USA; 3 Stealth BioTherapeutics, Newton, MA USA; 4 Massachusetts General Hospital, Boston, MA USA

**Keywords:** Mitochondrial disease, Resource utilization, Health care cost, Multiple sclerosis, Amyotrophic lateral sclerosis

## Abstract

**Background:**

Mitochondrial disease (MD) is a heterogeneous group of disorders characterized by impaired energy production caused by abnormal oxidative phosphorylation. Diagnosis of MD is challenging given the variability in how the disease can affect an individual’s neurologic, cardiovascular, ophthalmologic, or gastroenterological systems. This study describes the health care utilization and cost in patients diagnosed with MD.

**Methods:**

This study was a retrospective claims analysis based on data from the Truven Health Analytics MarketScan Database and Milliman’s Consolidated Health Cost Guidelines Sources Database. For the purpose of this study the diagnosis of MD was defined by ICD-9-CM (prior to October 2015), and ICD-10-CM (October 2015 or later), and included patients identified between January 1, 2008 to December 31, 2015. ICD-9-CM code of 277.87 (disorders of mitochondrial metabolism) and the ICD-10-CM codes of E88.40, E88.41, E88.42 and E88.49 (mitochondrial metabolism disorders) were used as inclusive criteria. Patients were included if they had at least six months of exposure after the first MD-related claim occurrence, and either one MD claim in the inpatient setting OR two MD claims in an outpatient setting. Claims of MD patients are compared to those of a general insured total member population, as well as to those from multiple sclerosis (MS) and amyotrophic lateral sclerosis (ALS) patients.

**Results:**

During the study period, 3825 patients between the ages of 0 and 15 (pediatric) and 4358 patients 16 years of age and greater (adult) were identified. Total allowed per member per month (PMPM) cost for pediatric patients was $4829 and $3100 for adults, compared with an average of $202 and $486, respectively, for the total member population. The greatest drivers of costs based on allowed claims came from inpatient, surgery, and prescription medications. In the adult population, MD imposes a PMPM cost burden that was comparable to that observed for multiple sclerosis ($3518) and ALS ($3460) patients.

**Conclusions:**

This retrospective claim study highlights the significant differences in the cost of medical care for MD patients compared to those of a general population. Mitochondrial disorders are associated with multisystem disease manifestations and a greater care and cost burden similar to other devastating neuromuscular diseases.

## Background

Mitochondrial disease (MD) is a group of rare genetic conditions caused by genetic mutations in the mitochondrial or nuclear genome encoding mitochondrial protein, resulting in impaired mitochondrial structure and function. Mitochondria are intracellular organelles that contain their own genetic material and are responsible for producing the majority of cellular energy. The defects in mitochondria are specific to the impairment of mitochondrial oxidative phosphorylation causing failure of mitochondrial function which results in cell injury and leads to cell death [1]. Secondary Mitochondrial Dysfunction (SMD) can be caused by factors influencing normal mitochondrial function such as non-mitochondrial related genetic conditions, specifically aging-associated genetic defects, environmental factors and other chronic conditions believed to be a principal source of reactive oxidative stress (ROS), resulting in mitochondrial damage.

MD affects approximately 1 in 5000 adults [2] and onset can occur at any age. Epidemiologic studies in MD have been challenging given that many patients with MD share features with other inherited or common conditions, such as diabetes mellitus, heart disease, neurodegenerative conditions or gastrointestinal diosrders which are common features of MD [3]. Additionally, diagnosis of MD is challenging due to the wide variability of symptoms and signs that a patient may have. Diagnosis may also be challenging given the lack of sensitive and specific biomarkers, as well as the cost and invasiveness of some tests, such as muscle biopsy, historically used for diagnosis. The gold standard for diagnosis of MD is to conduct genetic testing, although it continues to be an expensive process that is often not covered by health plans. However, improvements in the clinical and molecular profiling of MD [3] combined with the advancement of new molecular techniques are improving the diagnosis of MD. It remains that several patients may have a diagnosis of MD based only on clinical and/or biochemical findings, without an identifiable genetic mutation.

It is common for MD to present with multi-organ dysfunction with a progressive nature. Within MD, disease expression follows the principle of heteroplasmy in which each patient may have a different phenotypic expression despite the same genotype. The level of heteroplasmy, or the mixing of pathogenic and normal mitochondrial DNA in a cell contributes to variability in the clinical presentation of MD. Over the course of a lifetime, the ratio of normal and mutant mitochondrial DNA within a cell or tissue can change and helps explain severe presentations happening earlier in life (with high mutant load) and late-onset MD, where patients may be in generally good health until they develop signs of disease (lower mutant load). MD symptoms can also progress rapidly and may be exacerbated by viral illness, bacterial infection, other concomitant illness or surgery [4]. Due to this broad disease burden, MD patients tend to require multiple specialty physicians on their care team and are more susceptible to decompensations requiring medical interventions.

Supportive care is the mainstay of MD management, focusing on prevention of the exacerbation of symptoms during times of illness or physiologic stress [5]. There are currently no Food and Drug Administration (FDA) approved pharmacologic agents for the treatment of MD. Non-prescription agents used by patients include over-the-counter dietary supplements [6, 7] that are believed to improve Adenosine triphosphate (ATP) production and decrease free radical damage. However, there is little clinical evidence that shows these “mitochondrial cocktails” have a therapeutic benefit for the majority of MD patients. In fact, upwards of 38% of patients actually report improvements in symptoms following discontinuation of supplements [8].

A comprehensive and multidisciplinary care model is needed for the management of MD; however, very little is known about the health care resource utilization and cost associated with MD. This study was a retrospective database analysis of commercial claims data in the United States to understand (1) the drivers of resource utilization and cost in MD, and (2) how level of health resource utilization differs in MD compared to the general population and other well-studied neuromuscular disorders, such as multiple sclerosis (MS) and amyotrophic lateral sclerosis (ALS).

## Methods

### Data source

The data for this retrospective analysis are based on a commercially insured population, obtained from both the Truven Health Analytics MarketScan database and Milliman’s Consolidated Health Cost Guidelines Sources Database (CHSD) for the calendar years 2008 to 2015. Both sources of data represent patients receiving health insurance coverage through commercial means, including large employers, as well as individual, small- and large-group insurance carriers. Of note, other types of insurance coverage, including Medicaid and Medicare, are not included in this analysis. Given the complexity and chronic and debilitating nature of MD, the authors recognize that many patients ultimately may enroll in state and federally provided healthcare programs.

The Truven MarketScan database includes medical and drug claims data from employers and health plans, encompassing employees, their spouses, and dependents who are covered by employer-sponsored private health insurance. Information accessed included diagnostic codes, procedure codes, diagnosis-related grouping codes, National Drug Classification codes, site of service information, and the total amount paid through patient cost-sharing and by the payer, collectively referred to as the allowed amount. Milliman maintains administrative claims databases that link paid claims and encounter data that give detailed patient information across treatment sites and types of provider over time.

### Inclusion criteria

MD patients were identified through commercial claims based on the *International Classification of Disease 9th and 10th Revision* codes (ICD-9/− 10). Specifically, patients of any age with ICD-9277.87 (disorders of mitochondrial metabolism) or ICD-10 E88.40, E88.41, E88.42, and E88.49 (Table 1) were identified based on having at least 1 occurrence of the ICD-9 or ICD-10 code in the inpatient setting or 2 occurrences of the ICD-9 or ICD-10 code in any of the following settings: outpatient, professional, laboratory. MD patients were required to have at least 6 months of eligibility in the database after the occurrence of the first MD-related claim.

**Table 1:** Diagnosis codes used to identify mitochondrial disease

Diagnosis Codes Used to Identify Mitochondrial Disease	
ICD-9 277.87	Disorders of mitochondrial metabolism
ICD-10 E88.40 E88.41 E88.42 E88.49	Mitochondrial metabolism disorder, unspecified
	Mitochondrial Encephalomyopathy, Lactic Acidosis, and Stroke-like episodes (MELAS) syndrome
	Myoclonic Epilepsy with Ragged Red Fibers (MERRF) syndrome
	Other mitochondrial metabolism disorders

### Comparator group

The overall total member population cohort is derived from both the Truven MarketScan database and Milliman’s CHSD for the calendar year 2015 only. The overall baseline population includes 759,024,730 member months for 2015, or an average of 63,252,061 member years. The multiple sclerosis (MS) and amyotrophic lateral sclerosis (ALS) cohorts were used as comparator cohorts for other neuromuscular disorders. Both cohorts were also derived from both the Truven Marketscan database and Milliman’s CHSD for the calendar year 2015.

### Analyses

For resource utilization analysis, utilization values are reported on a claim per thousand basis, which represents the average annual claims utilization for a given service per one thousand patients. Cost figures are presented on an Allowed Per Member Per Month (PMPM) basis. Allowed dollars represents the total health care expenditures for a given service, comprising both patient cost sharing, as established in the patient’s plan of benefits, and the carrier’s paid dollars. Since the MD cohort spans from 2008 to 2015, an adjustment factor was applied to all MD allowed PMPMs to normalize costs to July 1, 2015 as an adjustment for medical inflation. This adjustment factor was based on medical trend factors from the Milliman’s Health Cost Guidelines. Allowed PMPMs for the total population, MS, and ALS cohorts are as of July 1, 2015. Comorbidities were identified based on diagnosis codes used by health care professionals during the member’s eligibility period.

## Results

### Patient characteristics

Of the patients who met the selection criteria for MD during the study period, the mean age of the study population (Table 2) was 26.1 years with 46.3% of patients aged 0 to 15 years (pediatric) and 53.7% aged 16 years and greater (adult). Males represented 46.8% (3832) and females represented 53.2% (4348) of the identified patients. A larger proportion of female MD patients (63.1%) were adults. In contrast, a larger proportion of male patients (57%) were of pediatric age. Of the adult patients, 62.5% were female.

**Table 2:** Patient demographic and clinical characteristics

Characteristics	MD Pediatric	MD Adult
Population, n (%)	3790 (46.3)	4390 (53.7)
Age^a^ in years, mean	7.4	42.3
Gender^a^, n		
Female (*n* = 4348) (%)	1605 (36.9)	2743 (63.1)
Male (*n* = 3832) (%)	2185 (57.0)	1647 (43.0)
Comorbidities, > 10% (%)		
	Seizure disorders and convulsions (47.4)	Diabetes (33.3)
	Asthma (30.2)	Asthma (22.6)
	Autistic disorder (25.4)	Seizure disorders and convulsions (22.5)
	Amyloidosis, porphyria, and other metabolic disorders (24.3)	Major depressive and bipolar disorders (18.3)
	Cerebral palsy, except quadriplegic (19.1)	Congestive heart failure (17.2)
	Down syndrome, Fragile X, other chromosomal anomalies, and congenital malformation syndromes (16.8)	Adrenal, pituitary, and other significant endocrine disorders (15.4)
	Disorders of the immune mechanism (16.4)	Specified heart arrhythmias (13.6)
	Spina Bifida, and other brain/spinal, nervous system congenital anomalies (16.3)	Acute pancreatitis/other pancreatic disorders and intestinal malabsorption (12.8)
	Cardio-respiratory failure and shock, including respiratory distress syndromes (15.3)	Amyloidosis, porphyria, and other metabolic disorders (11.8)
	Pervasive developmental disorders, except autistic disorder (12.5)	Cardio-respiratory failure and shock, including respiratory distress syndromes (10.4)
	Congestive heart failure (11.9)	Chronic obstructive pulmonary disease, including bronchiectasis (10.4)
	Parkinson’s, Huntington’s, and Spinocerebellar disease, and other neurodegenerative disorders (11.5)	
	Acute pancreatitis/other pancreatic disorders and intestinal malabsorption (11.3)	
	Atrial and ventricular septal defects, patent ductus arteriosus, and other congenital heart/circulatory disorders (10.2)	
	Myasthenia Gravis/myoneural disorders and Guillain-Barre syndrome/Inflammatory and toxic neuropathy (9.8)	
	Congenital/Developmental Skeletal and Connective Tissue Disorders (9.5)	

^a^3 patients did not have age or gender data available

Comorbidities in the pediatric and adult MD populations affect multiple bodily systems as shown in Table 2, which is typical of MD. There were 16 comorbid conditions that were present in > 10% of the MD pediatric population. Seven were neurological disorders, with seizure disorders and convulsion presenting in 47.4%, and autistic disorders presenting in 25.4% of MD pediatric patients. Respiratory diseases represented 2 out of the top 16 comorbidities, with asthma being present in 30.2% of MD pediatric patients. Immune disorders included disorders of the immune mechanism (16.4%) and myasthenia gravis/myoneural disorders and Guillain-Barre syndrome/inflammatory and toxic neuropathy (9.8%). Congestive heart failure (CHF) was present in 11.9% of patients, likely due to the high-energy demand of the cardiac muscle.

In the adult MD population there were 11 comorbid conditions that were present in > 10% of adult MD patients. Diabetes was the most prevalent condition, reported in 33% of MD adult patients. Three of the top 11 comorbidities were respiratory disorders, including asthma (22.6%), cardio-respiratory failure and shock (10.4%), and chronic obstructive pulmonary disease (10.4%). Neurological disorders present in > 10% of the adult MD patients included seizures disorders and convulsion (22.5%) and major depressive and bipolar disorders (18.3%). Top cardiac disorders included CHF (17.2%) and cardiac arrhythmias (13.6%). The high number of comorbidities speaks to the cost and burden of care associated with managing these multiple diagnoses.

Table 3 reports the percentages of all claims represented by each drug class. The most prescribed drug class for both pediatric and adult MD patients was anticonvulsants, representing 15.0% of all prescriptions for pediatrics and 10.7% of all prescriptions for adults. For the pediatric population, metabolic modifiers were the second most prescribed drug class, representing 8.0% of claims, while this drug class represented 2.2% of adult prescriptions. Opioid agonists were the second most prescribed class of drug for adults, representing 6.2% of prescriptions. In both pediatric and adult MD patient groups, proton pump inhibitors were the third most prescribed drug class, representing 4.0 and 3.3% in each group, respectively.

**Table 3:** Prescription drug classes, > 2.0% (%)

MD Pediatric	MD Adult
Anticonvulsants (15.0)	Anticonvulsants (10.7)
Metabolic modifiers (8.0)	Opioid agonists (6.2)
Proton pump inhibitors (4.0)	Proton pump inhibitors (3.3)
Sympathomimetics (2.3)	Thyroid hormone (2.9)
Selective serotonin reuptake inhibitors (2.0)	Selective serotonin reuptake inhibitors (2.7)
Antiadrenergic antihypertensives (2.0)	Metabolic modifiers (2.2)
	Central muscle relaxants (2.1)

### Health care cost and resource utilization

MD-related health care cost in comparison to the cost for the total member population is summarized in Table 4. The mean total MD allowed PMPM cost was $3958 ($455 per claim), which was significantly greater (*p* < 0.001) than the allowed PMPM cost for the total member population, with a mean of $427 ($206 per claim). These findings were consistent in both the pediatric and adult member population. Allowed PMPM cost for MD patients was higher for pediatric patients ($4829) than for adult patients ($3100), whereas in the total member population, the allowed PMPM cost was greater for the adults ($486) than pediatrics ($202). The average allowed per claim was approximately twice as much in the MD population compared to the total member population.

**Table 4:** Health care cost in the md population compared to the total member population

	Sample size (n)		Allowed PMPM*		Average per Claim	
	MD	Total Member	MD	Total Member	MD	Total Member
Pediatric	3794	13,029,839	$4829	$202	$511	$193
Adult	4389	50,222,222	$3100	$486	$390	$207
All Ages	8183	63,252,061	$3958	$427	$455	$206

*PMPM* per member per month, *p-value < 0.001

Health care resource utilization and cost were greater for MD patients than the total member population for all settings (Table 5). The most frequently used health resources in patients diagnosed with MD were prescription drugs (31%), physician visits (20%), and home health (10%). The top 3 greatest resources used in the total member population came from prescription drugs (41%), physician visits (20%), and laboratory testing (11%). The highest MD-related PMPM per claim were in the categories of inpatient hospitalizations ($769; 19%), surgery ($746; 19%), and prescription drugs ($582; 15%). Health care cost was higher for MD patients than for the total member population in all categories, with the exception of colonoscopy.

**Table 5:** Health care resource utilization and cost in the md population compared to the total member population

	Utilization per 1000 (%)		Allowed Claim PMPM (%)	
	MD	Total Member	MD	Total Member
Rx	32,338 (31)	10,198 (41)	$582 (15)	$102 (24)
Physician	20,500 (20)	5033 (20)	$306 (8)	$41 (10)
Home Health	10,247 (10)	226 (1)	$483 (12)	$5 (0)
Therapy	9655 (9)	949 (4)	$138 (3)	$8 (2)
DME	6514 (6)	302 (1)	$245 (6)	$5 (1)
Laboratory	7121 (7)	2736 (11)	$119 (3)	$17 (4)
Radiology	4213 (4)	1364 (5)	$91 (2)	$25 (6)
MH/SA	3146 (3)	544 (2)	$60 (2)	$9 (0)
Outpatient	2902 (3)	514 (2)	$183 (5)	$20 (5)
Surgery	2704 (3)	867 (3)	$746 (19)	$108 (25)
Preventative	1957 (2)	1305 (5)	$19 (0)	$13 (3)
Other	1042 (1)	302 (1)	$18 (0)	$2 (0)
ER	963 (1)	357 (1)	$85 (2)	$21 (5)
Inpatient	421 (0)	31 (0)	$769 (19)	$31 (7)
Ambulance	305 (0)	49 (0)	$35 (1)	$3 (1)
Genetic Testing	202 (0)	4 (0)	$40 (1)	$0 (0)
Muscle Biopsy	37 (0)	0 (0)	$3 (0)	$0 (0)
Colonoscopy	28 (0)	53 (0)	$1 (0)	$3 (1)
Maternity	21 (0)	54 (0)	$4 (0)	$15 (4)
Rehabilitation	9 (0)	1 (0)	$30 (1)	$1 (0)
Total	10,4324 (100)	24,889 (100)	$3958 (100)	427 (100)

*Rx* prescription drug, *DME* durable medical equipment, *MH/SA* mental health/substance abuse, *ER* emergency room

Pediatric MD patients utilized more physician specialties compared to the total member pediatric population as shown in Table 6. The top 3 physician specialties visited by MD pediatric patients were pediatric (24%), general practice (9%) and neurology (6%). The greatest increase in resource utilization in the physician setting for pediatric MD patients compared to the total member pediatric population was physical therapy (108.3-fold). Other specialties that showed a greater than 50-fold increase in utilization for MD patients were gastroenterology (61.0 fold), intensivist (56.5 fold), and neurology (52.0 fold).

**Table 6:** Resource utilization by physician specialty in the md population compared to the total member population

Pediatric MD			Adult MD		
	Count per 1000 (%)	Fold Increase – MD/Total Member		Count per 1000 (%)	Fold Increase – MD/Total Member
Pediatric	5054 (24)	5.4	Internal Medicine	2908 (15)	4.4
General Practice	1793 (9)	5.3	General Practice	2018 (10)	11.3
Neurology	1352 (6)	52.0	Family Practice	1814 (9)	2.0
Gastroenterology	1128 (5)	61.0	Neurology	1379 (7)	19.2
Physical Therapy	796 (4)	108.3	Cardiology	1117 (6)	3.8
Family Practice	787 (4)	2.8	Chiropractic	691 (3)	3.8
Surgery	747 (4)	8.2	Gastroenterology	580 (3)	7.8
Multispecialty Clinic	682 (3)	20.7	Pulmonary Disease	553 (3)	7.8
Cardiology	548 (3)	9.0	Emergency Medicine	412 (2)	8.6
Pulmonary Disease	480 (2)	30.3	Orthopedic Surgery	424 (2)	2.7
Emergency Medicine	439 (2)	20.4	Allergy / Immunology	386 (2)	3.8
Intensivist	357 (2)	56.5	Ophthalmology	362 (2)	2.8

Across all physician specialties, resource utilization was greater for the MD adult population compared to the total member adult population, as shown in Table 6. The top 3 physician specialties seen by MD patients were internal medicine (15%), general practice (10%) and family practice (9%). The greatest increase in resource utilization in the physician setting for MD compared to total member population was neurology (19.2-fold), which is consistent with the large proportion of neurologic comorbidities in the MD population (Table 2).

### Other neuromuscular disorders

**Fig. 1:**
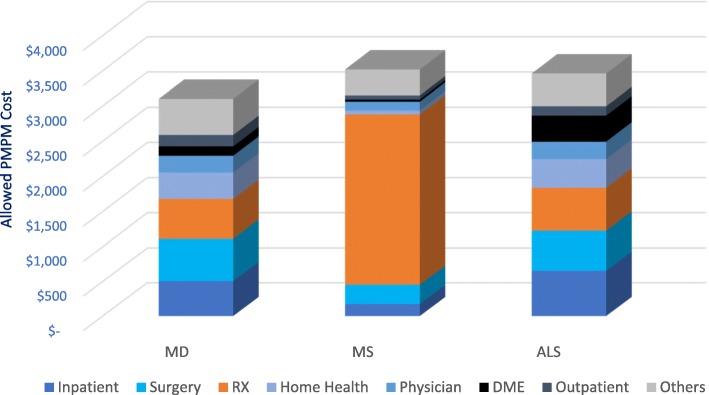
Comparison of health care resource utilization and cost among neuromuscular disorders in the adult population

Health care resource utilization and cost for adult MD patients were compared to those of 125,434 multiple sclerosis members and 4579 amyotrophic lateral sclerosis members for calendar year 2015 (Fig. 1). Resource utilization per 1000 for MD was 95,404, which was lower than ALS (107,133) but greater than MS (71,859). Overall MD allowed PMPM costs were comparable to MS and ALS, although the allowed PMPM per claim for MS was higher. Given the relatively small number of pediatric members with MS (*n* = 443) and ALS (*n* = 46), comparison of a pediatric cohort was not conducted.

**Fig. 2:**
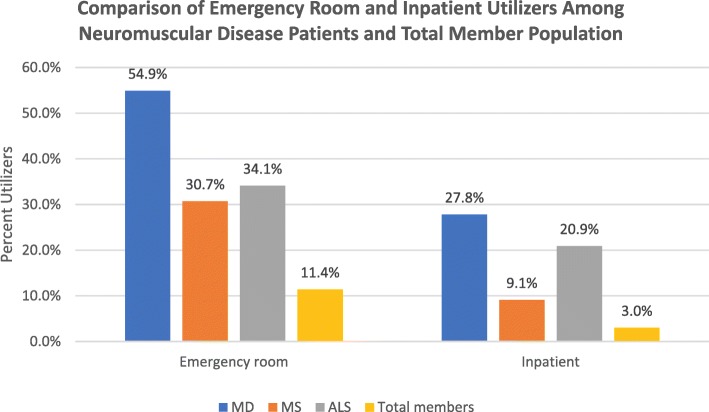
Comparison of emergency room and inpatient utilizers among neuromuscular disease patients and total member population

As shown in Fig. 1, the top 3 drivers of health care cost for MD and ALS patients were inpatient, surgery and prescription drugs. In contrast, the most significant expense for MS patients was prescriptions drugs (69%). As compared to the total population, MD, MS and ALS patients utilized the emergency room and inpatient resources at a higher percentage than the total member population (Fig. 2). A greater percentage of MD patients (54.9%) experienced an ER visit compared to MS (30.7%) or ALS (34.1%) patients. Inpatient visits were comparable in MD (27.8%) and ALS patients (20.9%) but both were greater than the 9.1% of MS utilizers in the inpatient setting.

## Discussion

This study conducted in a US patient population used ICD-9-CM and ICD-10-CM codes to identify MD patients in a large commercially insured database, representing approximately 63 million total members. Individuals were identified as having MD based on having at least 1 claim in the inpatient setting or at least 2 claims in the outpatient setting that contained the ICD code as described in the Methods. It is important to note that since these ICD codes do not capture molecular diagnosis, patients identified within this database may have either a primary or secondary MD. The data showed that the proportion of females (53%) and males (47%) with MD were comparable, although females with MD represented a larger proportion of the adult population (62.5% vs. 37.5%) than males with MD. This sex discrepancy might be related to adult females with maternal mitochondrial inheritance being at higher risk for developing symptoms compared to male parents. Additionally, females might be more afflicted with common complex comorbidities that are either misdiagnosed as MD or associated to secondary mitochondrial dysfunction (i.e. auto-immune disorders, fibromyalgia, autonomic dysfunction). MD may present at any age, although infantile diagnosis is associated with the greatest severity and likelihood of death. Children (mean age 7.4 years) make up just under half of the MD population. Given the chronic, complex and debilitating nature of the disease, MD patients of all ages are likely to rely on multiple healthcare providers and specialists, have an increased reliance on inpatient, emergency and surgical services, and are greater consumers of prescription drugs than the general population.

Clinical manifestations of MD can affect multiple bodily systems and often compromise more than one major organ concurrently. The comorbid conditions identified in MD patients in this study were based on diagnosis codes during the eligibility period before or after the diagnosis of MD and reflect the wide range of organ systems affected by MD. In both pediatric and adult patients, seizure disorders and convulsions were most common (47.4 and 22.5%, respectively), which is consistent with the most prescribed drug class, anticonvulsants, for both groups. The neurological manifestations are expected since the nervous system contains metabolically demanding cells that are highly ATP dependent [7]. Diabetes mellitus often arises as part of the clinical presentation associated with MD [9] and was present in 33.4% of adult MD patients (22.5% without complications and 10.9% with chronic complications) [10–13]. Mitochondrial dysfunction has been suggested as a potential pathogenic mechanism of airway disease, through oxidative stress and calcium deregulation [14]. It is thus interesting to note that 30% of identified pediatric and 22.6% of adult MD patients had asthma, which is higher than the 8% estimate for 2010 by the CDC [15] and 6 and 4% for the total member pediatric and adult population, respectively.

The cardiac muscle is a high consumer of energy with congestive heart failure present in 12% of pediatric MD patients compared to 0% in the total pediatric member population. CHF was present in 17% of adult MD patients compared to only 2% in the adult total member population. Another retrospective study in 200 patients with confirmed PMD also found cardiac involvement in 30% of patients [16]. The presence of multiple comorbid conditions suggests that MD merits particular attention in routine clinical care given the multiple manifestations in organ systems. In fact, recently published standards of care guidelines by the Mitochondrial Medicine Society recommend a thorough evaluation of the appropriate laboratory, cardiac, vision and developmental/cognitive evaluation at the time of diagnosis, annually, and as needed depending on symptoms or disease type [17].

We have found that health care resource utilization for MD patients are associated with serious illness, substantial health care resource utilization and cost compared to the general population. Total allowed PMPM cost for MD patients was $3958 compared to $427 for total member population. The higher cost is driven mainly by inpatient and surgery-related claims, both representing 19% of the allowed PMPM cost. The severity of illness for MD is such that hospitalization and surgeries for some patients are unavoidable. A previous study using the Kids’ Inpatient Database and the National Inpatient Survey from the Healthcare Cost and Utilization Project (HCUP) showed that hospitalizations for mitochondrial disease is associated with substantial costs estimated at $113 million in 2012 [18]. In our analysis we found that total health care cost for pediatric MD was greater than that of adult MD ($4829 vs. $3100). This finding may be due to the greater severity of disease in pediatric patients as compared to adults. The HCUP survey reported that in-hospital mortality was approximately 6-fold greater in MD children and approximately 3-fold greater in MD adults compared to the non-MD population within the same age group.

The scarcity of metabolic specialists highlights the need for both the primary care physician and specialists to recognize common clinical presentations of MD and evaluate or refer patients with suspicious symptoms for a further MD-specific diagnosis. Internal medicine, general practice and family practice represented 44% of the resource utilization in the physician setting for adult MD patients, with increases of 4.4-, 11.3-, and 2.0-fold over the general population, respectively. For pediatric MD patients, pediatrics, general practice, and neurology were the top 3 resource intensive physician specialty, representing 39% of total resource utilization with increases of 5.4, 5.3, and 52.0-fold over total pediatric members, respectively. The increase in resource utilization is a consequence of the management of the complex nature of MD.

Neurology represented the third most resource intensive specialty for pediatrics and exhibited the highest fold increase among top specialties for adults. This is reflective of the neurologic-associated symptoms and comorbidities of MD causing a variety of developmental and neurological disabilities [17]. Our data showed that neurologic disorders that were present in over 15% of MD pediatric patients were autism (25%), cerebral palsy (19%), and spinal bifida (16%). In the adult, SMD has also been implicated in other neurodegenerative diseases such as Alzheimer’s, Parkinson disease, ALS, and Huntington disease.

Other specialties with high utilization for MD compared to the general population were emergency medicine (20.4-fold in pediatrics and 8.6-fold in adults), pulmonary disease (20.3-fold in pediatrics and 7.8-fold in adults), and gastroenterology (61.0-fold in pediatrics and 7.8-fold in adults). This is consistent with the multi-systemic nature of MD affecting any organ with any degree of severity and requiring chronic as well as acute care through high increase in emergency medicine resources. The HCUP study showed that in children, emergency room visits were associated with higher in-hospital mortality [18]. Although the lungs are typically not directly involved in the clinical manifestation of MD, neuromuscular and CNS involvement may affect ventilation, both the ventilator drive as well as motor control, and mistakenly get classified as a primary pulmonary problem. Respiratory symptoms may include noisy breathing, snoring, obstructive apnea, central apnea, sleep disturbances, daytime hyper-somnolence and exercise intolerance due to ventilatory insufficiency, aspiration and infection. Gastrointestinal manifestations can include intestinal dysmotility, constipation, poor weight gain, swallowing dysfunction, and pancreatic and hepatic dysfunction [19]. MD patients are also at risk for liver dysfunction and ultimately, liver failure. Liver transplantation may be indicated in certain cases [8].

Additional specialties that exhibited high fold increases in resource utilization but were not among the most resource intensive practices were dietician and occupational therapist for pediatrics, representing almost a 200-fold increase over the non-MD cohort. MD symptoms may include insufficient caloric intake, poor appetite, and inadequate weight gain. MD patients require a comprehensive evaluation of their nutrition and potential deficiencies and may require alternative methods of feeding such as the use of gastric feeding through a gastrostomy tube, caloric supplementation, increased meal frequency, limited fasting, or intravenous nutrition. Occupational therapy can help children achieve targeted physical, cognitive and behavioral goals, as well as help diagnose and treat specific learning disabilities. In adults, pain management showed the highest fold resource use compared to the non-MD group, although it was not among the highest resource intensive specialty. Pain management is a component of mitochondrial patient care and includes management of headaches, neuropathic, muscle, and abdominal pain. Pain is recognized as being a more common feature of PMD than previously thought, this may explain the high utilization of opioid agonists in adults.

Although prescription drug cost is the largest driver of resource utilization, representing 31% of total health care resource use, its influence on cost for MD is only 15%, likely due to the lack of a FDA-approved prescription treatment for MD. Anticonvulsants were the most prescribed drug class for MD patients. Opioids and muscle relaxants represented 4 and 2% of prescription drugs for adult MD, respectively. Metabolic modifiers, which were the second most prescribed drug class for pediatrics, include several dietary supplements such as L-carnitine which serves as a mitochondrial shuttle for fatty acids, Coenzyme Q10; an electron acceptor and other vitamins necessary for proper mitochondrial function. The third most prescribed medication class for both pediatric and adult MD was proton pump inhibitors, which are typically used for gastroesophageal reflux disease [19]. Although gastroesophageal reflux disease (GERD) was not listed as one of the top comorbidities in our analysis, GERD as well as gastrointestinal complaints have been previously identified as top comorbid in-hospital diagnoses for admissions related to mitochondrial disease [17]. Smooth muscle cells of the intestines and gastrointestinal tract may be impaired due to myopathy similar to that found in muscle cells. Other cost of therapy not captured is the use of vitamins, nutritional supplements, and other over the counter medications.

ALS and MS were identified as two highly studied neurodegenerative disorders that carry a significant morbidity and mortality and were used for comparison to MD. Within the last 10 years, the multiple sclerosis community has seen approvals of several new medical therapies that include 4 monoclonal antibodies, 3 novel oral agents, and a pegylated version of interferon beta. In 2017, the FDA approved the first drug to treat ALS since 1995. However, there has yet to be an FDA approved drug for MD. We compared health care resource utilization for the three neuromuscular diseases in the adult population. Health care resource utilization for MD was lower than ALS, and both were greater than MS. MD costs were comparable with MS and ALS, although the average allowed per claim for MS was higher. MD patients experienced a higher percentage of visits to the ER and inpatient visits than MS or ALS patients. Inpatient and surgery claims each represented 19% of health care cost for MD patients and were the biggest drivers of cost, with prescription drug representing 15%. The top 3 drivers of health care cost were similar for ALS. A prospective study that evaluated the annual cost related to Friedreich’s Ataxia, a neuromuscular disorder due to a genetic mutation in the mitochondrial protein frataxin, showed that hospitalizations contributed to 21% of total direct medical costs in the UK [20], which is consistent with our finding. In MS, prescription drug was the single biggest driver of health care cost, as evident by the number and cost of disease modifying treatments approved for MS. It is difficult to accurately estimate the life-long MD cost given the heterogeneity of the disease with different ages of onset, symptoms severity, and prognosis. Life expectancy is likely higher than ALS and possibly similar to MS, and with an increasing number of patients with molecular diagnosis, the numbers and costs of MD will likely rise. Continued research in developing new therapies for MD will be key to avoid certain hospitalizations or surgeries while improving the quality of life for patients but will add to the health care prescription costs.

### Limitations

This study has several limitations. There are inherent faults in choosing the restrictive ICD codes. Given the relative rarity of diagnosis, there are likely patients with certain mitochondrial disorders that are never included in the database either because they have never been diagnosed with a mitochondrial disease, or the provider may not be familiar with the specific ICD terminology, or may have chosen a more general ICD code such as a code for myopathy or encepholopathy. This factor will result in patients with real mitochondrial diseases not being included in this data analysis. Conversely, because the choice of the ICD terminology does not require a high level of clinical certainty that a patient has MD, there could be patients included in the database that were suspected of having a MD but never had the clinical evidence to justify that choice of the specific ICD code. This issue has been outlined in recent publications [7]. We also recognize that the ICD codes may not capture the entire spectrum of MDs. Inclusion into this analysis required either 1 inpatient or 2 outpatient mitochondrial disease-specific claims and may have missed MDs with a legitimate single outpatient claim. Therefore, the MD cohort may have been underrepresented or have been preselected to identify a more severe patient population. In addition, mitochondrial disease is unique when compared to other well-studied neuromuscular disorders in three key ways: a) characterized by multisystem disease, b) MD has both acute and chronic aspects, with patients potentially living many years after diagnosis with minimal or progressive symptoms while simultaneously experiencing multiple acute exacerbations, c) there is not a uniform approach to disease management, leading to a variety in the types of prescription drugs and therapies prescribed, due to the overall heterogeneity of the disease. This analysis is limited to patients covered by private insurance plans. There may be inherent differences between these patients and patients covered by public insurance plans such as Medicare and Medicaid. The National Health Interview Survey 2017 reports that the percent of insured adults under 65 years is much higher under private plans (65.4%) as compared to public plans (25.3%). This may affect the generalizability of this analysis [21]. We did not analyze the differences in claims and utilization based on geographical areas/regions. This is because there are limited MD specialists and providers in the US, and most patients need to travel to the nearest specialist provider to receive treatment. Thus, conclusions from a geographical analysis would be driven by location of specialists rather than provisions of care based on state or region [22]. These variables contribute to the complexity of the disease and to the difficulties associated with a large population-based claims analysis.

## Conclusions

It is clear that mitochondrial pathology places a major burden on patients, caregivers, and the community. The clinical manifestation of MD on multiple major organs and systems is reflected by the significant representation of cost through inpatient hospitalizations and surgeries. These disorders are a major health issue, and appropriate resources are required to pursue research into effective treatments to address the consequence of the genetic defects as opposed to just alleviating symptoms. We have demonstrated that MD imposes a cost burden that is comparable to the more widely recognized neuromuscular disorders, MS and ALS, making it important for health plans to ensure that patients with MD receive the most appropriate care across multiple health disciplines.

## Abbreviations

ALS: Amyotrophic Lateral Sclerosis
ATP: Adenosine Triphosphate
CHF: Congestive Heart Failure
CHSD: Consolidated Health Cost Guidelines Sources Database
DME: Durable Medical Equipment
DNA: Deoxyribonucleic Acid
ER: Emergency Room
FDA: Food and Drug Administration
GERD: Gastroesophageal Reflux Disease
HCUP: Healthcare Cost and Utilization Project
ICD-10-CM: International Classification of Disease 10th Revision Codes
ICD-9-CM: International Classification of Disease 9th Revision Codes
MD: Mitochondrial Disease
MELAS Syndrome: Mitochondrial Encephalomyopathy, Lactic Acidosis, and Stroke-like episodes
MERRF Syndrome: Myoclonic Epilepsy with Ragged Red Fibers
MH/SA: Mental Health/Substance Abuse
MS: Multiple Sclerosis
PMD: Primary Mitochondrial Disease
PMPM: Per Member Per Month
ROS: Reactive Oxidative Stress
Rx: Prescription Drug
SMD: Secondary Mitochondrial Dysfunction
UK: United Kingdom
US: United States
